# Patient-Specific Predictive Modeling Using Random Forests: An Observational Study for the Critically Ill

**DOI:** 10.2196/medinform.6690

**Published:** 2017-01-17

**Authors:** Joon Lee

**Affiliations:** ^1^ Health Data Science Lab School of Public Health and Health Systems University of Waterloo Waterloo, ON Canada

**Keywords:** forecasting, critical care, predictive analytics, patient similarity, random forest

## Abstract

**Background:**

With a large-scale electronic health record repository, it is feasible to build a customized patient outcome prediction model specifically for a given patient. This approach involves identifying past patients who are similar to the present patient and using their data to train a personalized predictive model. Our previous work investigated a cosine-similarity patient similarity metric (PSM) for such patient-specific predictive modeling.

**Objective:**

The objective of the study is to investigate the random forest (RF) proximity measure as a PSM in the context of personalized mortality prediction for intensive care unit (ICU) patients.

**Methods:**

A total of 17,152 ICU admissions were extracted from the Multiparameter Intelligent Monitoring in Intensive Care II database. A number of predictor variables were extracted from the first 24 hours in the ICU. Outcome to be predicted was 30-day mortality. A patient-specific predictive model was trained for each ICU admission using an RF PSM inspired by the RF proximity measure. Death counting, logistic regression, decision tree, and RF models were studied with a hard threshold applied to RF PSM values to only include the M most similar patients in model training, where M was varied. In addition, case-specific random forests (CSRFs), which uses RF proximity for weighted bootstrapping, were trained.

**Results:**

Compared to our previous study that investigated a cosine similarity PSM, the RF PSM resulted in superior or comparable predictive performance. RF and CSRF exhibited the best performances (in terms of mean area under the receiver operating characteristic curve [95% confidence interval], RF: 0.839 [0.835-0.844]; CSRF: 0.832 [0.821-0.843]). RF and CSRF did not benefit from personalization via the use of the RF PSM, while the other models did.

**Conclusions:**

The RF PSM led to good mortality prediction performance for several predictive models, although it failed to induce improved performance in RF and CSRF. The distinction between predictor and similarity variables is an important issue arising from the present study. RFs present a promising method for patient-specific outcome prediction.

## Introduction

Harnessing the information contained in health data from various sources toward personalized medicine has been a research topic of interest and discussed by a number of highly regarded health researchers recently [1-5]. In particular, patient outcome prediction is an important topic in health care since accurate prognostic information can inform treatment planning and resource allocation. While prognostic scoring systems have traditionally been developed based on large population studies, the current rapid transition to electronic health records (EHRs) has led to an increased interest in data-driven, patient-specific outcome prediction models. Large-scale EHR data enable personalized predictive models where the degree of similarity between an index patient (for whom a prediction is to be made) and a past patient (the clinical data of whom can be found in an EHR repository) is taken into consideration. Such a personalized approach ensures that the predictive model is optimized for the index patient rather than the average patient in the population by building a customized predictive model just for the index patient.

A key to personalized patient outcome prediction is how patient similarity is defined. Various similarity measures have been investigated in the context of EHR-based outcome prediction, including distance-based [6,7] and cluster-based [8] methods. In addition, propensity score matching [9] employs a similar approach by identifying patients with similar likelihoods of receiving the treatment under investigation. A defined similarity measure is called a patient similarity metric (PSM), which can be calculated between patients. Subsequently, PSM values can be used to either discard the EHR patient data below a certain threshold or weight EHR patients’ contributions to predictive modeling proportionately to the PSM magnitude.

Outside of health, several domains have employed similarity approaches in machine learning and predictive analytics. One prime example is product recommendation in e-commerce where purchase histories of similar consumers are leveraged to recommend products to a given customer [10]. Furthermore, a variety of subspace clustering [11,12] and mixture models [13] have been developed and applied to identify and use similar cases across different application domains.

Our previous studies in this line of research investigated a cosine similarity PSM for personalized mortality prediction in intensive care unit (ICU) patients with hard thresholding [14] and bagging [15]. The results were promising and showed that using data from only similar patients, rather than the entire available data set, leads to better predictive performance. In order to study the effects of the particular choice of PSM on the results, however, it is worth investigating other PSMs.

One interesting way to define a PSM is to use the random forest (RF) proximity measure, which represents the likelihood of 2 cases falling in the same terminal node in the trees of an RF [16]. Xu and colleagues have developed the case-specific random forest (CSRF) using the RF proximity measure as a bootstrap sampling weight [17]. While this approach used RF proximity in personalizing the standard RF, the RF proximity can also be used as an independent PSM for predictive models other than the RF. Being a stochastic similarity measure, RF proximity is certainly distinct from the cosine similarity PSM we previously studied and as a result may capture patient similarity from a different perspective. Also, other similar approaches reviewed above (propensity score matching, subspace clustering, etc) tend to rely on well-known distance measures (such as the Euclidean or Mahalanobis) or clustering algorithms (such as K-means) to identify similar cases. To the best of our knowledge, an RF-inspired PSM has never been investigated for the purpose of patient outcome prediction in the ICU.

The objective of this study was to evaluate the effectiveness of RF patient similarity on improving mortality prediction performance in the critically ill.

## Methods

Patient data were extracted from the public ICU database Multiparameter Intelligent Monitoring in Intensive Care II (MIMIC-II) version 2.6 [18,19]. Because MIMIC-II is a deidentified, publicly available database, the need for a research ethics review was waived for this study. In order to make a direct comparison with our previous results [14,15], the same data set of 17,152 ICU admissions was analyzed. Table 1 lists the predictor variables extracted from MIMIC-II. The outcome variable was 30-day mortality. ICU admissions with missing data were excluded, and each included ICU admission was treated as an independent “patient,” as was done before.

**Table 1:** List of predictor variables.

Category	Predictor variables
Demographics	Age, gender
Administrative information	Admission type (elective, urgent, emergency), ICU^a^ service type (MICU^b^, SICU^c^, CCU^d^, CSRU^e^)
Vital signs (min. and max. every 6 hours during the first 24 hours in the ICU)	Heart rate, mean blood pressure, systolic blood pressure, SpO_2_, spontaneous respiratory rate, body temperature
Labs (min. and max. from the first 24 hours in the ICU	Hematocrit, white blood cell count, serum glucose, serum HCO_3_, serum potassium, serum sodium, blood urea nitrogen, and serum creatinine
Intervention (yes/no during the first 24 hours in the ICU)	Vasopressor therapy, mechanical ventilation, or continuous positive airway pressure
Others (from the first 24 hours in the ICU)	Worst Glasgow Coma Scale score, total urinary output every 6 hours

^a^ICU: intensive care unit.

^b^MICU: medical intensive care unit.

^c^SICU: surgical intensive care unit.

^d^CCU: coronary care unit.

^e^CSRU: cardiac surgery recovery unit.

In MIMIC-II, there are 1.24 ICU admissions per patient on average which indicates that most patients have only one ICU admission. Other admissions from the same patient are likely to contain useful and relevant information since they represent the most similar “patients” (determined by the PSM) for the index patient. This is akin to incorporating past patient history in most prognostic scoring systems. Most importantly, if the data from other admissions from the same patient are available at the time of prediction, it would be a waste of data if they are not taken into account.

Out of 29,149 adult ICU admissions in MIMIC-II, 17,152 (58.84%) had complete data and were included in the present study.

In the data set, the overall 30-day mortality rate was 15.10% (4,401/29,149), while 56.70% (16,527/29,149) were male. The average age was 64.5 years with a standard deviation of 17.0. The percentages of elective, urgent, and emergency admissions were 18.00% (5,247/29,149), 3.70% (1,078/29,149), and 78.30% (22,824/29,149), respectively. More detailed descriptive statistics of the data set can be found elsewhere [14]. All data were extracted from MIMIC-II using SQL Developer version 3.2.09 (Oracle Corp).

**Figure 1:**
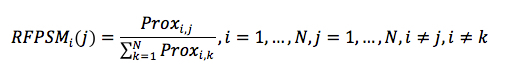
Random forest patient similarity metric formula.

Following how bootstrap sampling weights were calculated in CSRF [17], the RF PSM in the present study was calculated first by growing an RF in unsupervised mode using all data with an *mtry* value equal to the total number of predictor variables, a *nodesize* of 5, and 500 trees. When used in an unsupervised manner, RFs are capable of developing a dissimilarity measure among unlabeled data, which has been deployed for differentiating between observed and synthetic data [16,20]. The proximity values from this RF were normalized as displayed in Figure 1 to quantify the similarity between an index and another patient.

where *i* and *j* refer to the index and the *jth* patient in the data, respectively, and *Prox*_i,j_ is the number of trees in the grown RF that have both the index and *jth* patient in the same terminal node. This RF PSM was calculated for every pair of patients in the data.

For every index patient, the M most similar patients in the training data (ie, hard thresholding on RF PSM) were used to train a customized predictive model. A total of 4 different models were evaluated with hard thresholding: death counting (DC, predicted mortality risk is equal to the empirical probability of death among the M most similar patients), logistic regression (LR), decision tree (DT), and RF.

The range of M values varied depending on the predictive model. For DC, M ranged from 10 to 15,000 with a step size of 10, whereas for LR and RF the range was from 4000 to 15,000 with a step size of 1000. The M range for DT was from 5000 to 15,000 with a step size of 1000. This variation in M range accounted for computational burden and lack of variability in categorical variables (either predictor or outcome) among the M most similar patients when M was sufficiently small (only 1 category remained in some categorical variables when the M patients were too homogenous). Moreover, for LR, DT, and RF, training data size had to be sufficiently large (at least 1000), given that there were 75 predictor variables (Table 1). Since DC was associated with the least computational burden and was not subject to the issue of insufficient variability in categorical variables, it was evaluated with the widest M range with the smallest step size. The lower ends of the ranges for LR, DT, and RF were determined by trial and error. The step size of 1000 resulted in sufficient resolution allowing identification of predictive performance patterns, as will be evident in the Results section. In addition to the M ranges specified above, all training data were also used to represent traditional predictive modeling except for DC where using all training data implies using the overall mortality rate in the entire training data as the predicted mortality risk for all patients. In any case, 15,000 was very close to using all training data which included approximately 15,500 patients.

Note that we did not attempt to select the optimal M value for each patient, since our objective was to investigate the effects of the RF PSM on prediction performance as a function of M, as was done in our previous work [14].

As a fifth predictive model, CSRF was investigated. The entire data set was used for training CSRF models since the RF PSM was used as bootstrap sampling weights instead. Since this is a soft thresholding method, applying a range of M values was not applicable to CSRF.

Note that while DC, LR, and DT were investigated previously [14], RF and CSRF were not. RF and CSRF were included in this study in the spirit of conducting a comprehensive RF investigation in personalized predictive analytics.

Predictive performance was evaluated using the area under the receiver operating characteristic curve (AUROC) and the area under the precision-recall curve (AUPRC). A 10-fold cross-validation was conducted for all models to avoid overfitting. In each iteration of the cross-validation, the RF PSM between each patient in the test data and each patient in the training data was computed. Then, for each patient in the test data, the predictive models described above were trained using the M most similar cases in the training data (except CSRF), where M was varied as explained above. Once all patients in the test data were predicted, the AUROC and AUPRC for that fold were computed. Hence, the 10 iterations of the cross-validation yielded 10 AUROCs and 10 AUPRCs.

All computation was conducted in R version 3.3.1 (R Foundation). In particular, the *randomForest* package [21] was used to build RFs (with the default parameter values except for the unsupervised RF for proximity measure calculation, for which the parameter values were described above). CSRF models were constructed using the R code supplied by Xu et al [17], with a *nodesize* of 1 and 500 trees, while *mtry* was set to the same default value as the *randomForest* package (ie, floor of the square root of the number of predictors). LR and DT models were created using the *stats* and *rpart* packages, respectively, with the default parameter settings.

## Results

**Figure 2:**
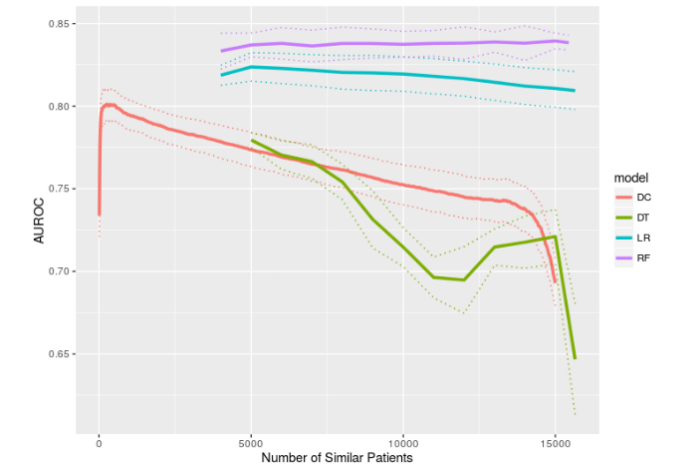
Mortality prediction performance measured in area under the receiver operating characteristic curve as a function of the number of similar patients. Mean and 95% confidence interval from 10-fold cross-validation are shown.

**Figure 3:**
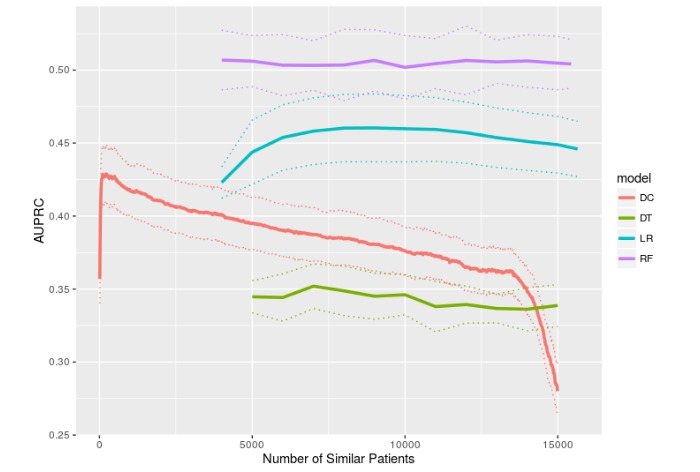
Mortality prediction performance measured in area under the precision-recall curve as a function of the number of similar patients. Mean and 95% confidence interval from 10-fold cross-validation are shown.

Figures 2 and 3 show the predictive performances of DC, LR, DT, and RF with hard thresholding, in terms of AUROC and AUPRC, respectively, as a function of the number of similar patients included in model training (ie, M). The pattern of exhibiting suboptimal performances when M is too small (due to small sample size) or too large (due to dissimilar patients being included in the training data) and a peak performance somewhere in the middle was most prominent in DC and mildly visible in LR in terms of both AUROC and AUPRC. Interestingly, DT showed 2 local maxima in Figure 2 and weakly showed 1 peak in Figure 3. RF did not seem to benefit from hard thresholding on RF PSM as its performance was relatively independent of M.

DC and DT showed statistically significant improvement (via 2-sided *t* tests) between the best performance (in terms of mean AUROC or AUPRC) and when the maximum number of patients were used as training data, with respect to both AUROC (DC: *P*<.001; DT: *P*<.001) and AUPRC (DC: *P*<.001; DT: *P*<.001). LR and RF showed statistically significant performance improvement for neither AUROC (LR: *P*=.07; RF: *P*=.75) nor AUPRC (LR: *P*=.36; RF: *P*=.85).

**Figure 4:**
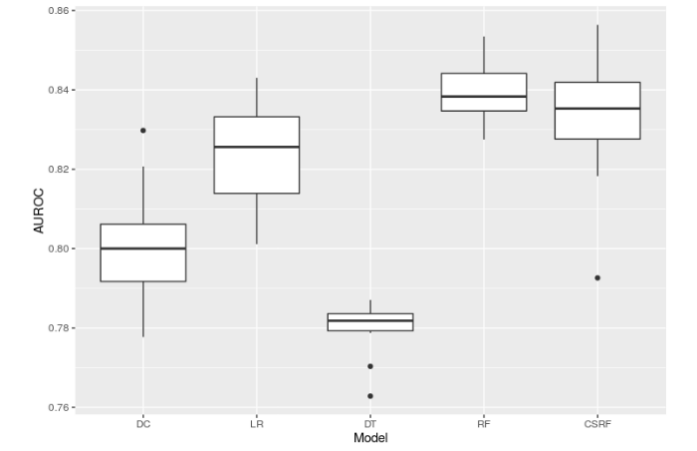
Box plot comparing area under the receiver operating characteristic curves (AUROCs) from all 5 models. For death counting, logistic regression, decision tree, and random forest, the performance from the number of similar patients corresponding to the maximum mean AUROC is shown.

**Figure 5:**
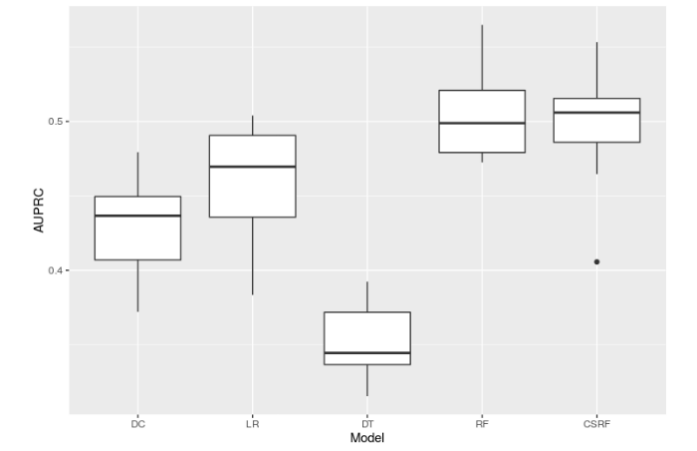
Box plot comparing area under the precision-recall curves (AUPRCs) from all 5 models. For death counting, logistic regression, decision tree, and random forest, the performance from the number of similar patients corresponding to the maximum mean AUPRC is shown.

Table 2 tabulates the best predictive performance of each model with respect to mean AUROC and AUPRC. The numbers of similar patients at which the best performance occurred as well as the performance of CSRF are also reported in Table 2. Figures 4 and 5 (AUROC and AUPRC) are boxplots that correspond to the best performances shown in Table 2 and enable a quick visual comparison among all 5 models. Note that these best performances simply correspond to the maximum mean AUROC or AUPRC and do not represent statistically significant peak performances, as evident in Figures 2 and 3. Overall, RF and CSRF resulted in the best performances, followed by LR, DC, and DT in decreasing order of performance.

**Table 2:** Best predictive performance from each random forest patient similarity metric (PSM) model in terms of mean area under the receiver operating characteristic curve and area under the precision-recall curve in comparison with cosine PSM and traditional models with no PSM. All cosine PSM results are from Lee et al [14].

	Number of similar patients at best predictive performance				Best predictive performance, mean (95% CI)					
	AUROC^a^		AUPRC^b^		AUROC			AUPRC		
	RF^c^ PSM^d^	Cosine PSM	RF PSM	Cosine PSM	RF PSM	Cosine PSM	No PSM	RF PSM	Cosine PSM	No PSM
DC^e^	260	100	230	60	0.801 (0.792-0.811)	0.797 (0.791-0.803)	0.693 (0.679-0.707)	0.429 (0.409-0.449)	0.393 (0.378-0.407)	0.280 (0.263-0.297)
LR^f^	5000	6000	9000	6000	0.824 (0.815-0.832)	0.830 (0.825-0.836)	0.811 (0.799-0.821)	0.460 (0.437-0.484)	0.474 (0.460-0.488)	0.449 (0.430-0.468)
DT^g^	5000	2000	7000	4000	0.779 (0.775-0.784)	0.753 (0.742-0.764)	0.721 (0.705-0.738)	0.352 (0.337-0.367)	0.347 (0.335-0.358)	0.339 (0.324-0.353)
RF	15000	—	4000	—	0.839 (0.835-0.844)	—	0.839 (0.835-0.844)	0.507 (0.527-0.486)	—	0.505 (0.487-0.523)
CSRF^h^	—	—	—	—	0.832 (0.821-0.843)	—	—	0.496 (0.520-0.471)	—	—

^a^AUROC: area under the receiver operating characteristic curve.

^b^AUPRC: area under the precision-recall curve.

^c^RF: random forest.

^d^PSM: patient similarity metric.

^e^DC: death counting.

^f^LR: logistic regression.

^g^DT: decision tree.

^h^CSRF: case-specific random forest.

## Discussion

### Principal Findings

The conventional doctrine in machine learning is that it is always beneficial to collect more training data. This is true if collected data represent the same underlying phenomenon, but it is often difficult to make this assumption in medicine largely due to enormous variability in patient/clinical characteristics, as well as our limited understanding of complex human health and disease pathways. In the era of big data, we can now afford to be more selective regarding which cases should be included in predictive modeling. Data-driven patient similarity matching, via a PSM, leads to objective training data selection and uses hidden patterns in multidimensional data that are difficult for human clinicians to identify.

In comparison with conventional, one-size-fits-all predictive models such as those from the Framingham Heart Study [22], patient-specific predictive models improve predictive performance at the cost of increased computational burden associated with computation of all pairwise PSM values and training of a unique custom model for each patient. This is a reasonable trade-off today given that powerful computing is available at ever falling prices. With big data analytics leveraging parallel computing, it is feasible to train and use patient-specific models in real time at the point of care.

In this study, RF and CSRF, which were not studied in our previous work, outperformed DC, LR, and DT, while the difference between RF and CSRF was statistically insignificant. The comparison between RF with CSRF is interesting because the difference is essentially hard thresholding versus weighted bootstrapping. Also, CSRF did not improve upon the conventional RF that used all data as training data. The comparable performances from RF and CSRF, and the negligible effects of the number of similar patients on RF performance (Figures 2 and 3), imply that not all predictive models benefit from the use of a PSM. This could be a characteristic of ensemble models but needs further research to clarify.

An appropriate method to circumvent the lack of variability in categorical variables when M is too small should be investigated in future work. One simple solution is to exclude such problematic categorical variables in model training after “using them up” for similarity matching. For example, gender may be used for PSM calculation but if the training data subsequently only include 1 gender (same as that of the index patient), then gender can be dropped from model training to avoid the computational issue. This solution will not work for the outcome variable, however. This issue is closely related to the important topic of how best to use the variables in a given data set in personalized predictive analytics: should they be used as predictor or similarity variables, or both? This topic requires further research involving both real life and simulated data.

Although this study only investigated prediction performance as a function of M without attempting to select optimal M values for individual patients, optimal M selection will become important when the results of this study are taken to practice. However, in the context of patient similarity, selecting the optimal M for a given patient is not trivial because it may vary across different types of patient. This implies that optimal M values should not be selected based on Figures 2 and 3 because they represent the cohort as a whole and individual patients may not follow the global patterns shown in Figures 2 and 3. For this reason, a subset of similar patients would have to be compiled first (assuming that they are sufficiently similar to yield similar optimal M values), and then this subset would have to be partitioned into training, validation, and test data so that the validation data can be used for M selection. The main challenge with this approach is that insufficient sample size is likely to occur, especially given that there were 75 predictor variables in this study. Furthermore, it is difficult to determine how many similar patients should be included in the subset and how similar they need to be to the patient under consideration.

Instead of selecting the M most similar patients from training data, it is also feasible to threshold RF PSM values so that all patients with an RF PSM above this threshold are included in model training. One advantage of this approach is that the quality of similarity in the training data can be ensured and controlled, whereas a fixed M would force M patients to be used for model training regardless of how similar they are to the index patient in absolute sense. However, a challenge with this approach is that a good understanding of the magnitude of the RF PSM is required, which could be the subject of another research study. Moreover, the threshold is likely to vary across patients.

In addition to the future work mentioned above, future PSM research directions should involve the following: other PSMs especially those that can capture temporal patterns, other health data sets, patient outcomes other than mortality, other predictor variables (such as diagnosis, medications other than vasopressors, information from free-text clinician notes, etc), and other predictive models.

### Comparison With Prior Work

In comparison with the DC, LR, and DT results from our previous work that studied a cosine similarity PSM [14], the predictive performance patterns as a function of the number of similar patients were similar in this study. In terms of the best performance of each model, 2 performances were better than the corresponding performances in our previous work (ie, no overlap between the 95% confidence intervals): DC and DT in terms of AUPRC and AUROC, respectively. The rest of the reported best performances did not show any significant difference between the 2 studies. Despite the modest performance improvement, these results indicate that the RF PSM outperformed the cosine similarity PSM.

However, the number of similar patients associated with the best performance was different between the 2 studies. The RF PSM tended to require more similar patients to be included in the training data to achieve peak performance; the cosine similarity PSM results peaked with respect to AUROC and AUPRC at 100 and 60 for DC, 6000 and 6000 for LR, and 2000 and 4000 for DT. This finding suggests that the RF and cosine similarity PSMs quantified patient similarity somewhat differently.

### Limitations

This study has limitations. First, MIMIC-II is a single-center database and hence the results may not be generalizable to other ICU data. Second, no extensive investigation of various parameter values was conducted for the predictive models in order to keep the number of models that had to be trained at a reasonable level. Although the default parameter settings from the widely used R packages are reasonable choices, the effects of the parameter values on predictive performance could be investigated further in future work.

### Conclusions

PSM-driven predictive analytics is an exciting topic, as accurate, tailor-made patient outcome prediction can greatly inform risk stratification and resource allocation. The results from this study that stem from the use of an RF PSM corroborate the utility of PSMs in enhancing predictive performance at the patient level. The superior predictive performances of RF and CSRF, as well as the fact that the RF PSM outperformed the cosine similarity PSM in some models, indicate that RFs are well suited for patient-specific predictive modeling.

## Abbreviations

AUPRC: area under the precision-recall curve
AUROC: area under the receiver operating characteristic curve
CCU: coronary care unit
CSRF: case-specific random forest
CSRU: cardiac surgery recovery unit
DC: death counting
DT: decision tree
EHR: electronic health record
ICU: intensive care unit
LR: logistic regression
MICU: medical intensive care unit
MIMIC II: Multiparameter Intelligent Monitoring in Intensive Care II
PSM: patient similarity metric
RF: random forest
SICU: surgical intensive care unit
